# Supplementary figures and images for: Targeting Hypoxia and HIF1α in Triple-Negative Breast Cancer: New Insights from Gene Expression Profiling and Implications for Therapy
Source: Biology (Basel). 2024 Jul 31;13(8):577. doi: 10.3390/biology13080577 (PMC11351882; doi:10.3390/biology13080577)

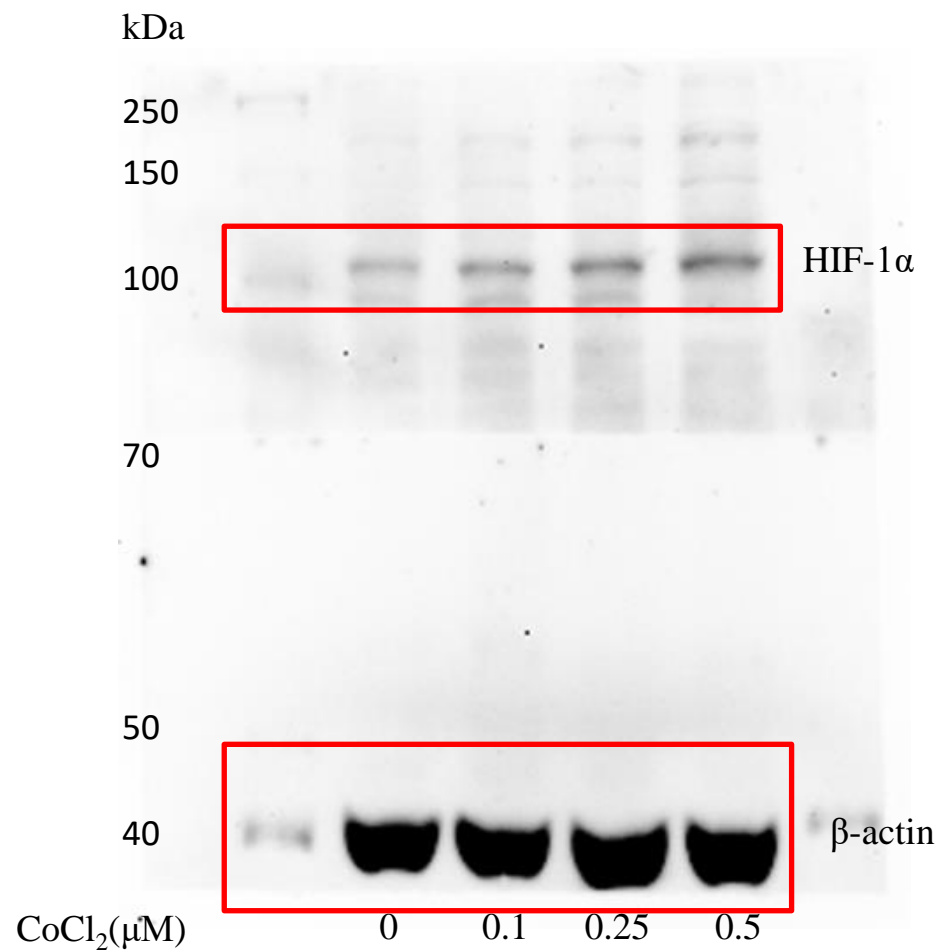

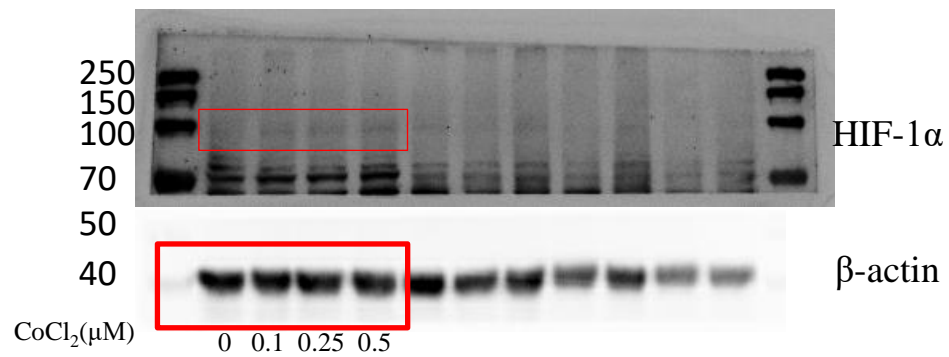

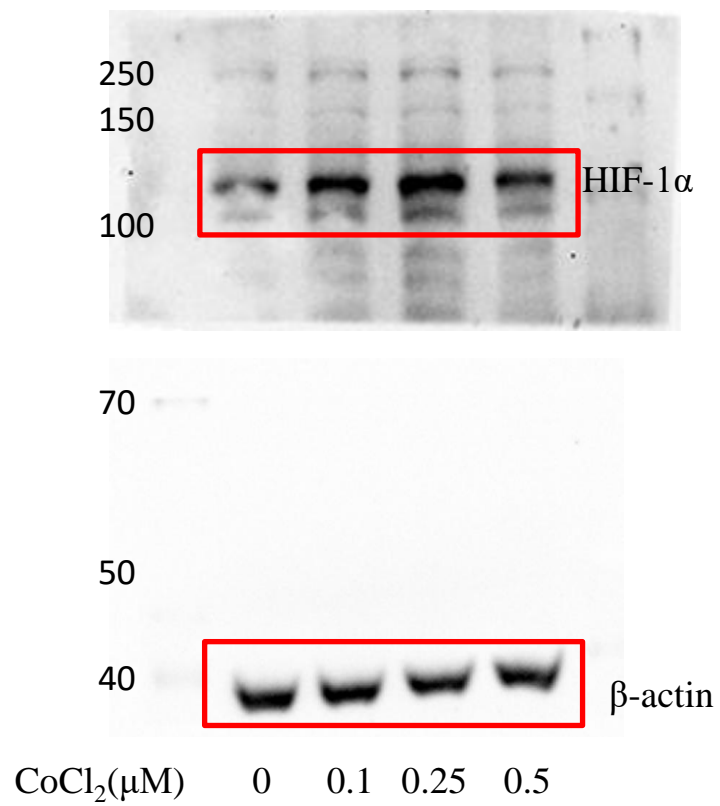

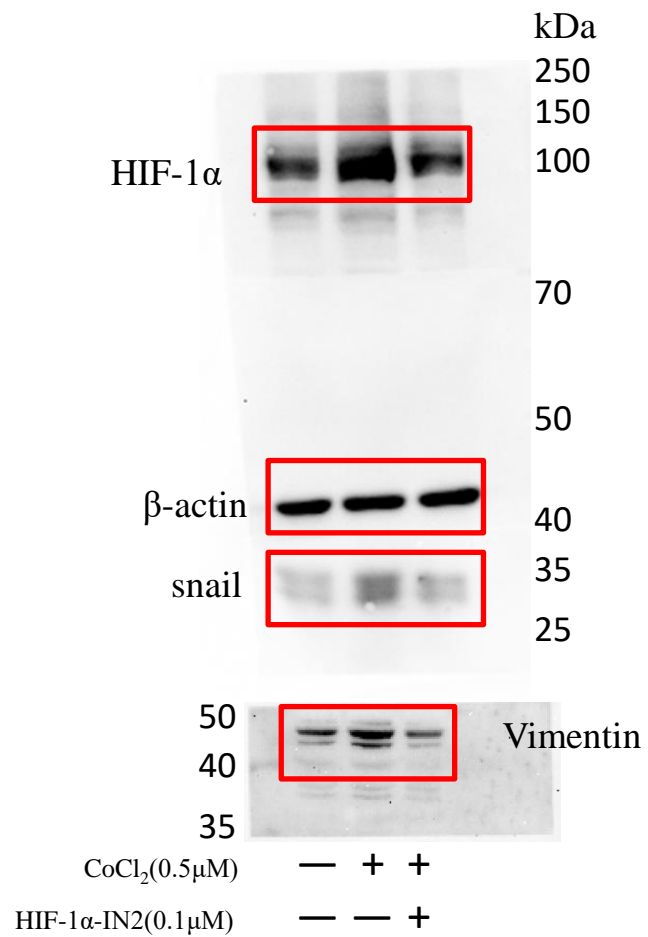

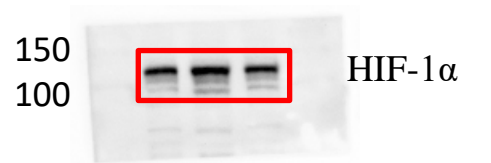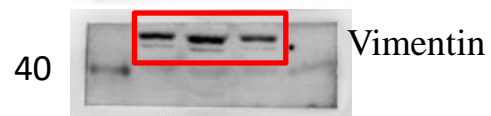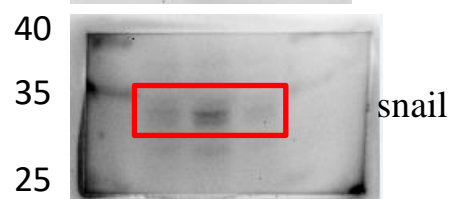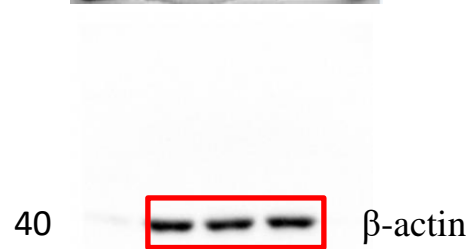

CoCl<sub>2</sub>(0.5 $\mu$ M)

— + +

HIF-1 $\alpha$ -IN2(0.1 $\mu$ M)

— — +

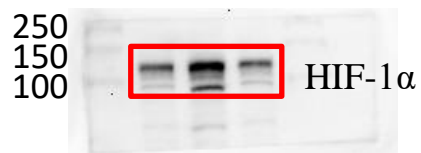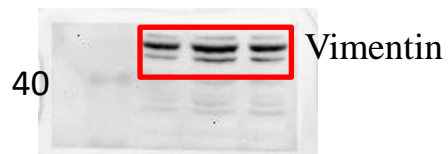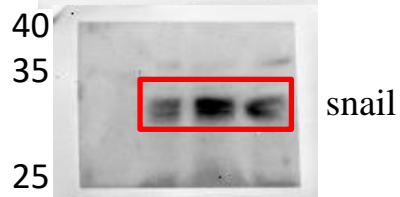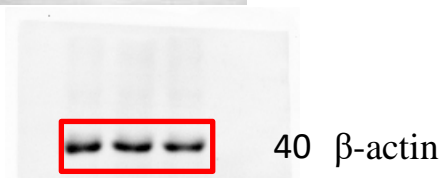

|                                  |   |   |   |
|----------------------------------|---|---|---|
| CoCl <sub>2</sub> (0.5 $\mu$ M)  | — | + | + |
| HIF-1 $\alpha$ -IN2(0.1 $\mu$ M) | — | — | + |

Supplement: Supplementary file 1 [file biology-13-00577-s001.zip › Figure S2.pdf]
